# A Study of Latent State-Trait Theory Framework in Piecewise Growth Models

**DOI:** 10.1177/01466216251360565

**Published:** 2025-07-15

**Authors:** Ihnwhi Heo, Ren Liu, Haiyan Liu, Sarah Depaoli, Fan Jia

**Affiliations:** 1 Department of Psychological Sciences, 33244 University of California, Merced, CA, USA

**Keywords:** latent state-trait theory, measurement theory, piecewise growth model, psychometrics, reliability, repeated measurement

## Abstract

Latent state-trait (LST) theory provides a psychometric framework that facilitates the measurement of long-term trait change and short-term state variability in longitudinal data. While LST theory has guided the development and extension of linear latent growth models within its theoretical framework, the integration of piecewise growth models (PGMs) into the LST theory framework remains uninvestigated. PGMs are well suited for modeling nonlinear developmental processes comprised of distinct stages, which frequently arise in psychological and educational research. Their ability to capture phase-specific changes makes them a useful tool for applied and methodological researchers. This paper introduces a novel measurement approach that integrates PGMs into the framework of LST theory by presenting single-indicator piecewise growth models (SI-PGMs) and multiple-indicator piecewise growth models (MI-PGMs). We detail the model specifications for both SI-PGMs and MI-PGMs. For SI-PGMs, we define the reliability coefficient; for MI-PGMs, we define the consistency coefficient, occasion specificity coefficient, and reliability coefficient. We then conduct simulations to evaluate the models’ performance in accurately recovering growth parameters and capturing true reliability. The simulation results indicated that SI-PGMs and MI-PGMs successfully recovered growth parameters and performed comparably in the absence of situational influences. However, MI-PGMs outperformed SI-PGMs when situational influences were present. We conclude by outlining directions for future research and providing M*plus* syntax to support the dissemination of the models.

Understanding the dynamics of psychological constructs through longitudinal data is a cornerstone of psychometric research, yet a critical issue exists: Measurement of psychological constructs cannot be fully separated from situational influences. Here, situational influences refer to transient conditions or external factors that can affect individuals’ thoughts, behaviors, or feelings at a given time of measurement. Latent state-trait (LST) theory provides a framework for decomposing variability in observed data into components attributable to traits, states, and measurement errors (Geiser, 2020; Steyer et al., 1999, 2015). Integrating latent growth models into the framework of LST theory is particularly effective for capturing how short-term state variability deviates from the overall trait level, while concurrently modeling trait change over time. The accuracy of measurement can be increased by taking into account interplays between traits and situational influences. As such, applying the LST theory framework allows for a more reliable interpretation of repeated measurements and enables a more nuanced understanding of underlying longitudinal processes. These advantages have promoted the widespread methodological application of LST theory within the context of linear latent growth modeling (Geiser, Bishop, et al., 2013; Geiser et al., 2015; Geiser, Keller, & Lockhart, 2012).

In psychological and educational measurement, overall change patterns in repeated measurements are often described by segmented growth over time. Piecewise growth models (PGMs), a special type of latent growth model, offer flexibility in describing nonlinear growth curves through separate growth phases. By specifying change points—called knots—to reflect stage-specific growth rates, PGMs provide more relevant information about the phasic change process. Recent years have witnessed a growing body of advancements in extending and evaluating PGMs (Depaoli et al., 2023; Diallo & Morin, 2015; Heo, Jia, & Depaoli, 2024, 2025; Kohli & Harring, 2013; Liu & Perera, 2023). Despite these contributions, the measurement aspect of PGMs has received relatively little attention. We highlight how incorporating PGMs within the LST theory framework helps address the challenge of accurately quantifying trait and state variability in segmented growth phases, a concern overlooked in current PGM applications. In addition, researchers can capture the reliability of measurements and improve the interpretability of phasic changes across both individual growth phases and the entirety of measurement occasions.

The primary objective of this paper is to bridge the gap between the theoretical potential of LST theory and its practical application to PGMs by addressing the measurement of trait changes and short-term state variability surrounding piecewise growth trends. Our methodology builds upon Bishop et al. (2015), Geiser, Bishop, et al. (2013), and Geiser, Keller, and Lockhart (2013) that utilized either single or multiple indicators for the formulation of models. Relatedly, we extend the multiple-indicator approach of Steyer et al. (1997) by modeling nonlinear growth trajectories that unfold across distinct phases. Compared to Steyer et al. (1997), our framework introduces latent trait change factors that link the latent state variables across measurement occasions, enabling the simultaneous decomposition of trait and state components. In doing so, we explicitly incorporate a knot component to model phase transitions and formally define and compute LST theory-based coefficients (i.e., consistency, occasion specificity, and reliability). Our contributions offer a novel measurement approach that integrates PGMs into LST theory, and we aim to encourage the implementation of these methods in applied research settings.

The remaining sections of this paper are structured as follows. We first introduce the basics of LST theory. We then define two PGMs based on the LST theory framework. This is followed by a simulation study to evaluate the performance of the proposed models. We conclude with a short discussion regarding the avenues for future research. Additionally, we provide M*plus* syntax as a resource for researchers.

## Fundamentals of Latent State-Trait Theory

This section outlines the fundamental and core concepts of LST theory. For a more detailed explanation, readers may consult Geiser (2020) and Steyer et al. (2015). In LST theory, several latent variables are defined to quantify the components of traits, state residuals, and measurement errors. First, the latent state variable ( $\tau_{it}$ ) is defined as the conditional expectation of an observed indicator $y_{it}$ , given the person and the situation variables:

(1) $$\tau_{it}≔E\left(y_{it}|P_{t},S_{t}\right).$$

In this definition, i denotes the $i^{\text{th}}$ indicator, and t represents a time point. The terms $P_{t}$ and $S_{t}$ respectively denote the person and the situation variables at time t . Here, $P_{t}$ and $S_{t}$ are random variables, whose values represent persons in situations at a given time. Thus, a value of the latent state variable equals that person’s hypothetical intraindividual mean in a particular situation at a given time. Next, the random measurement error variable ( $\epsilon_{it}$ ) is defined:

(2) $$\epsilon_{it}≔y_{it}-\tau_{it},$$

which is the difference between an individual’s measured observed variable and the latent state variable. The latent trait variable ( $\xi_{it}$ ) is then defined as the conditional expectation of an observed variable given the person variable at time t (i.e., $P_{t}$ ):

(3) $$\xi_{it}≔E\left(y_{it}|P_{t}\right).$$

A value of the latent trait variable is the intraindividual (true) mean of a person-at-time- t , where the person-at-time- t represents a realized value of the random variable $P_{t}$ . Lastly, the latent state residual variable ( $\zeta_{it}$ ) captures the deviation of an individual’s latent state scores from the latent trait scores:

(4) $$\zeta_{it}≔\tau_{it}-\xi_{it}.$$

The latent state residual variable represents the systematic influence of situations that shift latent state scores from latent trait scores. Therefore, the presence of differences between an individual’s state and trait scores indicates the presence of situational effects. Following the formulations of four latent variables, an observed indicator $y_{it}$ can be decomposed as follows:

(5) $$y_{it}=\tau_{it}+\epsilon_{it}=\xi_{it}+\zeta_{it}+\epsilon_{it}.$$

The right-hand side of Equation (5) indicates that a measured variable is the sum of the latent trait variable, the latent state residual variable, and the measurement error variable. Through this decomposition, contributions of stable traits to measurements are isolated from situation-specific variations.

## Integration of Piecewise Growth Models into the LST Theory Framework

In PGMs, a knot location is an important parameter that indicates the time point at which one growth phase transitions to another. This change reflects a shift in the slope of the growth trajectory. By specifying this knot, researchers can divide a longitudinal process into multiple phases, each with its own rate of change. Because the knot acts as an additional parameter, PGMs add complexity to model estimation when determining how and where the slope changes. In addition, the number of measurement occasions is tied to model estimation and identification (Bollen & Curran, 2006; Flora, 2008; Heo et al., 2025). According to Bollen and Curran (2006), at least five measurement occasions are needed to identify a PGM with two linear phases if the knot is positioned at the third time point. Having more measurement occasions additionally provides information for a more precise estimation of phase-specific slopes.

PGMs can be specified with a range of complexities. However, the linear-linear specification, defined by a single knot that is assumed to exist at the population level and segments growth trajectories into two distinct linear phases, remains among the most widely used in both methodological (e.g., Depaoli et al., 2023; Heo, Jia, & Depaoli, 2024, 2025; Kohli & Harring, 2013; Kwok et al., 2010; Leite & Stapleton, 2011; Ning & Luo, 2017) and applied research (e.g., Chung et al., 2017; Heo et al., 2024; Jaggars & Xu, 2016; Kohli & Sullivan, 2019; Li et al., 2019; Patrick & Schulenberg, 2011). These extant studies and applications suggest a widespread utility due to the interpretative clarity and indicate that this linear-linear form with a single knot characterizes typical findings. Note that while PGMs and their variants can be formulated with multiple knots (for example methodological works, see Harring et al., 2021; Heo, Jia, & Depaoli, 2024; Lock et al., 2018), our primary objective here is not to exhaustively explore multiple-knot specifications for PGMs within the LST theory framework. Rather, given the utility, interpretability, and prevalence of linear-linear trends, we focus specifically on this basic form of PGM to clearly illustrate how PGMs can be incorporated into the LST theory framework and to lay a foundation for further extensions. In the following paragraphs, we show how to specify PGMs in the framework of LST theory.

### Single-Indicator Piecewise Growth Model

**Figure 1.**
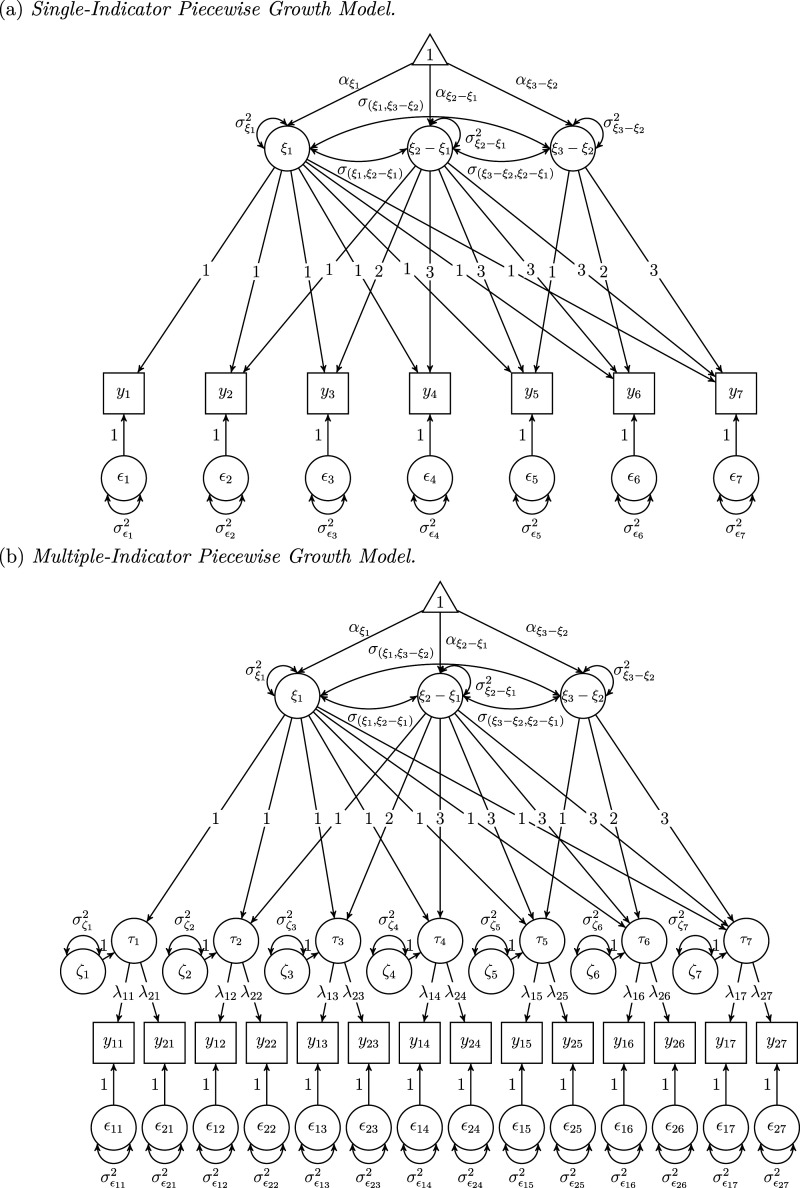
Example Path Diagrams. (A) Single-Indicator Piecewise Growth Model. (B) Multiple-Indicator Piecewise Growth Model

We first present the single-indicator piecewise growth model (SI-PGM). In SI-PGMs, single indicators are used to measure latent constructs. Therefore, SI-PGMs assume that psychological constructs of interest are strictly trait-like and do not depend on situational influences. Any changes observed are attributable to changes in traits only. A path diagram illustrating an example SI-PGM, with seven measurement occasions and a knot located at the fourth occasion, is provided in Figure 1(A). An SI-PGM is formulated as follows:

(6) $$y_{t}=\xi_{1}+\min\left(t_{j},t_{\gamma }\right)\left(\xi_{2}-\xi_{1}\right)+\max\left(t_{j}-t_{\gamma },0\right)\left(\xi_{3}-\xi_{2}\right)+\epsilon_{t},$$

where $\xi_{1}$ is the initial trait level, $\xi_{2}-\xi_{1}$ is the first trait change, $\xi_{3}-\xi_{2}$ is the second trait change, $\epsilon_{t}$ is the error term, $t_{j}$ is the time metric referring to a time point j (e.g., $t_{1}$ is equal to 0 because the first time point is usually coded as 0), and $t_{\gamma }$ is the time metric for the knot location (e.g., $t_{\gamma }$ is equal to 3 because the fourth occasion is the knot location). Note that the subscript i is omitted because only a single indicator is taken into account in SI-PGMs. Given this model definition, the variance of an observed indicator can be decomposed in the following manner:

(7) $$\text{var}\left(y_{t}\right)=\sigma_{\xi_{1}}^{2}+{\left\{\min\left(t_{j},t_{\gamma }\right)\right\}}^{2}\sigma_{\xi_{2}-\xi_{1}}^{2}+{\left\{\max\left(t_{j}-t_{\gamma },0\right)\right\}}^{2}\sigma_{\xi_{3}-\xi_{2}}^{2}+2\;\min\left(t_{j},t_{\gamma }\right)\sigma_{\left(\xi_{1},\xi_{2}-\xi_{1}\right)}+2\;\min\left(t_{j},t_{\gamma }\right)\max\left(t_{j}-\gamma ,0\right)\sigma_{\left(\xi_{2}-\xi_{1},\xi_{3}-\xi_{2}\right)}+2\;\max\left(t_{j}-t_{\gamma },0\right)\sigma_{\left(\xi_{1},\xi_{3}-\xi_{2}\right)}+\sigma_{\epsilon_{t}}^{2},$$

where $\sigma^{2}$ and σ , respectively, correspond to the variances of and covariances between factors, as indicated by their subscripts.

LST theory introduces several coefficients such as reliability, consistency, and occasion specificity. These coefficients quantify the degree of variability attributable to the precision of measurements, trait effects, and situational influences, respectively. When there is only a single indicator, short-term state variability cannot be separated from trait change and random measurement errors. For this reason, in SI-PGMs, the only coefficient that can be defined is the reliability ( $\text{rel}\left(y_{t}\right)$ ) coefficient:

(8) $$\text{rel}\left(y_{t}\right)=\frac{\begin{array}{l} \sigma_{\xi_{1}}^{2}+{\left\{\min\left(t_{j},t_{\gamma }\right)\right\}}^{2}\sigma_{\xi_{2}-\xi_{1}}^{2}+{\left\{\max\left(t_{j}-t_{\gamma },0\right)\right\}}^{2}\sigma_{\xi_{3}-\xi_{2}}^{2}+2\;\min\left(t_{j},t_{\gamma }\right)\sigma_{\left(\xi_{1},\xi_{2}-\xi_{1}\right)}\\ +2\;\min\left(t_{j},t_{\gamma }\right)\max\left(t_{j}-t_{\gamma },0\right)\sigma_{\left(\xi_{2}-\xi_{1},\xi_{3}-\xi_{2}\right)}+2\;\max\left(t_{j}-t_{\gamma },0\right)\sigma_{\left(\xi_{1},\xi_{3}-\xi_{2}\right)} \end{array}}{\text{var}\left(y_{t}\right)}.$$

The reliability coefficient, which ranges from 0 to 1, quantifies the proportion of measured score variance attributable to systematic sources of variability rather than unsystematic measurement error components. Higher values of the reliability coefficient indicate higher measurement precision.

### Multiple-Indicator Piecewise Growth Model

The second model we define is the multiple-indicator piecewise growth model (MI-PGM). In MI-PGMs, multiple indicators are incorporated, which makes it possible to further decompose observed variability to situational effects. Figure 1(B) describes a path diagram for an MI-PGM with two indicators per measurement occasion across seven repeated measurements, with a knot located at the fourth occasion. The formulation of MI-PGMs is as follows:

(9) $$y_{it}=\omega_{it}+\lambda_{it}\xi_{1}+\lambda_{it}\;\min\left(t_{j},t_{\gamma }\right)\left(\xi_{2}-\xi_{1}\right)+\lambda_{it}\;\max\left(t_{j}-t_{\gamma },0\right)\left(\xi_{3}-\xi_{2}\right)+\lambda_{it}\zeta_{t}+\epsilon_{it},$$

where $\omega_{it}$ is an additive term to satisfy the unidimensionality assumption in LST theory (Steyer, 1989), and $\lambda_{it}$ refers to the factor loadings of the $i^{\text{th}}$ indicator from latent state variables to observed variables at time j . Compared to SI-PGMs, MI-PGMs include the subscript i to denote multiple indicators. We can thus decompose the variance of an observed indicator as:

(10) $$\text{var}\left(y_{it}\right)=\lambda_{it}^{2}\sigma_{\xi_{1}}^{2}+\lambda_{it}^{2}{\left\{\min\left(t_{j},t_{\gamma }\right)\right\}}^{2}\sigma_{\xi_{2}-\xi_{1}}^{2}+\lambda_{it}^{2}{\left\{\max\left(t_{j}-t_{\gamma },0\right)\right\}}^{2}\sigma_{\xi_{3}-\xi_{2}}^{2}+2\lambda_{it\;}^{2}\;\min\left(t_{j},t_{\gamma }\right)\sigma_{\left(\xi_{1},\xi_{2}-\xi_{1}\right)}+2\lambda_{it}^{2}\;\min\left(t_{j},t_{\gamma }\right)\max\left(t_{j}-t_{\gamma },0\right)\sigma_{\left(\xi_{2}-\xi_{1},\xi_{3}-\xi_{2}\right)}+2\lambda_{it}^{2}\;\max\left(t_{j}-t_{\gamma },0\right)\sigma_{\left(\xi_{1},\xi_{3}-\xi_{2}\right)}+\lambda_{it}^{2}\sigma_{\zeta_{t}}^{2}+\sigma_{\epsilon_{it}}^{2}.$$

With multiple indicators, we can define two additional coefficients—consistency and occasion specificity—alongside reliability to quantify trait effects and situational effects. The consistency ( $\text{con}\left(y_{it}\right)$ ) coefficient reflects the degree of trait effects:

(11) $$\text{con}\left(y_{it}\right)=\frac{\begin{array}{l} \lambda_{it}^{2}\sigma_{\xi_{1}}^{2}+\lambda_{it}^{2}{\left\{\min\left(t_{j},t_{\gamma }\right)\right\}}^{2}\sigma_{\xi_{2}-\xi_{1}}^{2}+\lambda_{it}^{2}{\left\{\max\left(t_{j}-t_{\gamma },0\right)\right\}}^{2}\sigma_{\xi_{3}-\xi_{2}}^{2}\\ +2\lambda_{it}^{2}\;\min\left(t_{j},t_{\gamma }\right)\sigma_{\left(\xi_{1},\xi_{2}-\xi_{1}\right)}+2\lambda_{it}^{2}\;\min\left(t_{j},t_{\gamma }\right)\max\left(t_{j}-t_{\gamma },0\right)\sigma_{\left(\xi_{2}-\xi_{1},\xi_{3}-\xi_{2}\right)}\\ +2\lambda_{it}^{2}\;\max\left(t_{j}-t_{\gamma },0\right)\sigma_{\left(\xi_{1},\xi_{3}-\xi_{2}\right)} \end{array}}{\text{var}\left(y_{it}\right)}.$$

The consistency coefficient ranges from 0 to 1, and higher values mean that a measure reflects more trait influences. Next, the occasion specificity ( $\text{os}\left(y_{it}\right)$ ) coefficient quantifies situational influences:

(12) $$\text{os}\left(y_{it}\right)=\frac{\lambda_{it}^{2}\sigma_{\zeta_{t}}^{2}}{\text{var}\left(y_{it}\right)}.$$

The occasion specificity represents the proportion of indicator variance attributable to situational effects. The occasion specificity also ranges from 0 to 1, with higher values indicating that a measure reflects more situational influences. Finally, the reliability ( $\text{rel}\left(y_{it}\right)$ ) coefficient quantifies the degree of measurement precision:

(13) $$\text{rel}\left(y_{it}\right)=\frac{\begin{array}{l} \lambda_{it}^{2}\sigma_{\xi_{1}}^{2}+\lambda_{it}^{2}{\left\{\min\left(t_{j},t_{\gamma }\right)\right\}}^{2}\sigma_{\xi_{2}-\xi_{1}}^{2}+\lambda_{it}^{2}{\left\{\max\left(t_{j}-t_{\gamma },0\right)\right\}}^{2}\sigma_{\xi_{3}-\xi_{2}}^{2}\\ +2\lambda_{it}^{2}\;\min\left(t_{j},t_{\gamma }\right)\sigma_{\left(\xi_{1},\xi_{2}-\xi_{1}\right)}+2\lambda_{it}^{2}\;\min\left(t_{j},t_{\gamma }\right)\max\left(t_{j}-t_{\gamma },0\right)\sigma_{\left(\xi_{2}-\xi_{1},\xi_{3}-\xi_{2}\right)}\\ +2\lambda_{it}^{2}\;\max\left(t_{j}-t_{\gamma },0\right)\sigma_{\left(\xi_{1},\xi_{3}-\xi_{2}\right)}+\lambda_{it}^{2}\sigma_{\zeta_{t}}^{2} \end{array}}{\text{var}\left(y_{it}\right)}.$$

The reliability coefficient is the total proportion of true score variability and is interpreted in the same way in SI-PGMs described earlier. Note that reliability is the sum of consistency and occasion specificity.

## Simulation Study

### Design

We conducted a simulation study to evaluate the performance of SI-PGM and MI-PGM in recovering growth parameters and true reliability. Accordingly, we examine how well these models capture underlying piecewise growth patterns while assessing measurement properties. Our design included three factors: sample sizes, the magnitude of situational effects, and the analysis models. The sample sizes were 100, 250, and 500 to represent a range from small to large sizes common in simulation studies using PGMs (Depaoli et al., 2023; Heo, Jia, & Depaoli, 2024). This range also includes the median sample size observed in applications of LST theory (Geiser & Lockhart, 2012). For the magnitude of situational influences, we referred to Geiser, Keller, and Lockhart (2013) and manipulated the occasion specificity coefficients across each time point to be 0, 0.10, 0.25, and 0.40 to reflect no, small, medium, and large situational effects. The analysis models were the SI-PGM and MI-PGM displayed in Figure 1. We fully crossed all levels of the factors, resulting in 3 (sample sizes) × 4 (magnitude of situational effects) × 2 (analysis models) = 24 cells. We used M*plus* version 8.7 (Muthén & Muthén, 1998) to generate 1000 replications per cell and fitted analysis models to datasets using maximum likelihood estimation.

The data-generating model was the MI-PGM in Figure 1(B) which allowed us to manipulate the magnitude of situational effects. We derived the population values from Depaoli et al. (2023) and Geiser, Keller, and Lockhart (2013) and have provided them in the Online Supplemental Material^1^. In order to conveniently determine parameter values and compute reliabilities, we assumed all indicators to be tau-parallel and constrained the covariances between growth factors to be zero (Geiser, Keller, & Lockhart, 2013; Leite, 2007). The true reliability of each indicator in the MI-PGM was set to be 0.8; for fitting the SI-PGM, we aggregated the two indicators by obtaining the arithmetic mean of two indicators at each measurement occasion. Following the Spearman-Brown formula (Eisinga et al., 2013), the reliability of the aggregated indicators in the SI-PGM was 0.889.

The outcome measure was the relative bias for growth parameters and reliability. We computed relative bias as the difference between the estimated and true values of growth parameters or reliability, relative to the true values. When the true value was 0, we only calculated the absolute difference between the estimates and the true values. For the relative bias of reliability, we computed the bias for each indicator based on its respective true reliability, according to the analysis model in the simulation conditions.

## Results

Growth parameter and reliability estimates for all models were obtained without any estimation issues across replications. We first report the recovery of growth parameters, followed by that of reliability for both model types.

Table 1 displays the simulation results for the relative bias of growth parameters. Across all conditions, no substantial bias was observed, as all relative bias values fell within the range of −0.1 to 0.1. While no bias was evident in that regard, the relative bias of growth parameter variances appeared larger than that of other parameters when the sample size was 100, though it approached closer to 0 as the sample size increased. These results indicate that the proposed SI-PGM and MI-PGM successfully recovered the growth parameters, ensuring that piecewise growth curves were accurately captured when the knot was positioned at the fourth time point.

**Table 1.** Relative Bias in Growth Parameter Estimates

	Population value	100				250				500			
		No	Small	Medium	Large	No	Small	Medium	Large	No	Small	Medium	Large
Single-indicator piecewise growth model													
$E\left(\xi_{1}\right)$	2.5	<0.001	<0.001	<0.001	<0.001	<0.001	<0.001	<0.001	<0.001	<0.001	<0.001	<0.001	<0.001
$E\left(\xi_{2}-\xi_{1}\right)$	0.5	0.001	0.001	0.001	0.001	0.001	0.001	0.001	0.001	<0.001	<0.001	<0.001	0.001
$E\left(\xi_{3}-\xi_{2}\right)$	0.75	0.001	0.001	0.001	0.001	<0.001	<0.001	<0.001	<0.001	<0.001	<0.001	<0.001	<0.001
$\sigma_{\xi_{1}}^{2}$	0.2	−0.011	−0.012	−0.014	−0.017	−0.005	−0.005	−0.006	−0.007	−0.001	−0.001	<0.001	0.003
$\sigma_{\xi_{2}-\xi_{1}}^{2}$	0.1	−0.011	−0.012	−0.015	−0.020	−0.005	−0.005	−0.005	−0.007	−0.001	−0.001	−0.001	−0.001
$\sigma_{\xi_{3}-\xi_{2}}^{2}$	0.1	−0.017	−0.021	−0.029	−0.042	−0.006	−0.006	−0.007	−0.008	−0.003	−0.002	−0.001	0.002
$\sigma_{\left(\xi_{1},\xi_{2}-\xi_{1}\right)}$	0	<0.001	<0.001	0.001	0.001	<0.001	<0.001	<0.001	0.001	<0.001	<0.001	<0.001	<0.001
$\sigma_{\left(\xi_{1},\xi_{3}-\xi_{2}\right)}$	0	<0.001	<0.001	<0.001	<0.001	<0.001	<0.001	<0.001	<0.001	<0.001	<0.001	0.001	0.001
$\sigma_{\left(\xi_{2}-\xi_{1},\xi_{3}-\xi_{2}\right)}$	0	<0.001	<0.001	<0.001	<0.001	<0.001	<0.001	<0.001	<0.001	<0.001	<0.001	<0.001	<0.001
Multiple-indicator piecewise growth model													
$E\left(\xi_{1}\right)$	2.5	<0.001	<0.001	<0.001	<0.001	<0.001	<0.001	<0.001	<0.001	<0.001	<0.001	<0.001	<0.001
$E\left(\xi_{2}-\xi_{1}\right)$	0.5	0.001	0.001	0.001	0.001	0.001	0.001	0.001	0.001	<0.001	0.001	0.001	0.001
$E\left(\xi_{3}-\xi_{2}\right)$	0.75	0.001	0.001	0.001	0.002	<0.001	<0.001	<0.001	0.001	<0.001	<0.001	<0.001	<0.001
$\sigma_{\xi_{1}}^{2}$	0.2	−0.011	−0.012	−0.014	−0.017	−0.004	−0.005	−0.005	−0.006	−0.001	<0.001	0.001	0.003
$\sigma_{\xi_{2}-\xi_{1}}^{2}$	0.1	−0.011	−0.011	−0.014	−0.020	−0.004	−0.004	−0.004	−0.006	−0.001	<0.001	<0.001	−0.001
$\sigma_{\xi_{3}-\xi_{2}}^{2}$	0.1	−0.017	−0.021	−0.030	−0.046	−0.005	−0.005	−0.006	−0.006	−0.002	−0.001	<0.001	0.003
$\sigma_{\left(\xi_{1},\xi_{2}-\xi_{1}\right)}$	0	<0.001	<0.001	0.001	0.001	<0.001	<0.001	<0.001	0.001	<0.001	<0.001	<0.001	<0.001
$\sigma_{\left(\xi_{1},\xi_{3}-\xi_{2}\right)}$	0	<0.001	<0.001	<0.001	<0.001	<0.001	<0.001	<0.001	0.001	<0.001	<0.001	0.001	0.001
$\sigma_{\left(\xi_{2}-\xi_{1},\xi_{3}-\xi_{2}\right)}$	0	<0.001	<0.001	<0.001	<0.001	<0.001	<0.001	<0.001	<0.001	<0.001	<0.001	<0.001	<0.001

*Note.* The column headings of “No,” “Small,” “Medium,” and “Large” represent the magnitude of situational influences, corresponding to the variances of state residual factors set at 0, 0.1, 0.25, and 0.4, respectively. Relative bias values reported as “< 0.001” indicate that the absolute value of the bias was smaller than 0.001, regardless of whether the original bias was positive or negative.

Table 2 presents the simulation results for the relative bias in recovering true reliability. To aid interpretation, we bolded values of relative bias exceeding 10%. Overall, both the SI-PGM and MI-PGM performed similarly in successfully recovering true reliability when situational influences were absent across all conditions. However, differences emerged in conditions with the presence of situational influences. Even a small degree of situational effects resulted in substantial bias in the SI-PGM, such that bias intensified systematically as the magnitude of situational effects increased. Under large situational influences, the bias underestimated true reliability by approximately 50%. This consistent pattern across all measured time points highlights the sensitivity of the SI-PGM to situational effects. In contrast, the MI-PGM exhibited negligible bias and maintained recovery of true reliability irrespective of the magnitude of situational effects. Relative biases remained minimal (<1%) across varying degrees of situational influences and time points.

**Table 2.** Relative Bias in Reliability Estimates

	True reliability	100				250				500			
		No	Small	Medium	Large	No	Small	Medium	Large	No	Small	Medium	Large
Single-indicator piecewise growth model													
$y_{1}$	.889	−0.002	**−0.128**	**−0.317**	**−0.504**	<0.001	**−0.126**	**−0.313**	**−0.501**	<0.001	**−0.124**	**−0.311**	**−0.498**
$y_{2}$	.889	−0.003	**−0.129**	**−0.317**	**−0.503**	−0.002	**−0.127**	**−0.314**	**−0.500**	−0.001	**−0.126**	**−0.312**	**−0.499**
$y_{3}$	.889	−0.003	**−0.130**	**−0.318**	**−0.505**	−0.001	**−0.127**	**−0.314**	**−0.514**	<0.001	**−0.125**	**−0.312**	**−0.499**
$y_{4}$	.889	−0.003	**−0.130**	**−0.318**	**−0.506**	−0.001	**−0.126**	**−0.314**	**−0.501**	−0.001	**−0.126**	**−0.313**	**−0.500**
$y_{5}$	.889	−0.002	**−0.128**	**−0.314**	**−0.501**	−0.001	**−0.126**	**−0.313**	**−0.500**	<0.001	**−0.125**	**−0.312**	**−0.499**
$y_{6}$	.889	−0.004	**−0.131**	**−0.321**	**−0.508**	−0.001	**−0.127**	**−0.315**	**−0.502**	<0.001	**−0.125**	**−0.312**	**−0.500**
$y_{7}$	.889	−0.004	**−0.132**	**−0.323**	**−0.511**	−0.002	**−0.128**	**−0.317**	**−0.504**	−0.001	**−0.127**	**−0.314**	**−0.501**
Multiple-indicator piecewise growth model													
$y_{11}$	.800	−0.001	0.001	0.003	0.004	−0.001	<0.001	<0.001	<0.001	<0.001	<0.001	<0.001	0.001
$y_{21}$	.800	−0.004	−0.004	−0.004	−0.004	−0.002	−0.001	−0.001	−0.001	−0.001	−0.001	−0.001	−0.001
$y_{12}$	.800	−0.002	−0.001	0.001	0.002	<0.001	0.001	0.002	0.002	0.001	0.001	0.001	0.002
$y_{22}$	.800	−0.003	−0.003	−0.003	−0.002	−0.001	−0.001	−0.001	−0.002	<0.001	−0.001	−0.001	−0.001
$y_{13}$	.800	−0.006	−0.004	−0.003	−0.002	−0.002	−0.001	−0.001	<0.001	−0.001	<0.001	<0.001	<0.001
$y_{23}$	.800	−0.007	−0.006	−0.005	−0.004	−0.002	−0.002	−0.002	−0.001	−0.001	−0.001	−0.001	−0.001
$y_{14}$	.800	−0.005	−0.004	−0.002	−0.001	−0.002	−0.002	−0.001	−0.001	−0.001	−0.001	<0.001	<0.001
$y_{24}$	.800	−0.004	−0.002	−0.001	<0.001	−0.002	−0.002	−0.001	−0.001	−0.001	−0.001	−0.001	−0.002
$y_{15}$	.800	−0.004	−0.003	−0.002	−0.002	−0.002	−0.001	−0.001	−0.001	−0.001	<0.001	<0.001	<0.001
$y_{25}$	.800	−0.004	−0.002	−0.002	−0.001	−0.002	−0.001	−0.001	−0.001	−0.001	<0.001	<0.001	−0.001
$y_{16}$	.800	−0.005	−0.004	−0.002	<0.001	−0.002	−0.001	<0.001	0.001	−0.001	−0.001	<0.001	<0.001
$y_{26}$	.800	−0.002	−0.001	0.001	0.002	−0.002	−0.001	−0.001	−0.001	−0.001	−0.001	−0.001	−0.001
$y_{17}$	.800	−0.004	−0.003	−0.002	<0.001	−0.001	<0.001	0.001	0.001	<0.001	<0.001	0.001	0.001
$y_{27}$	.800	−0.004	−0.003	−0.002	−0.001	−0.002	−0.002	−0.001	−0.001	<0.001	<0.001	<0.001	<0.001

*Note.* Relative bias exceeding 10% was bolded. The column headings of “No,” “Small,” “Medium,” and “Large” represent the magnitude of situational influences, corresponding to the variances of state residual factors set at 0, 0.1, 0.25, and 0.4, respectively. Relative bias values reported as “< 0.001” indicate that the absolute value of the bias was smaller than 0.001, regardless of whether the original bias was positive or negative.

Sample sizes showed negligible influence on the relative bias within both analysis models. For the SI-PGM, bias patterns related to the magnitude of situational effects remained consistent across different sample sizes (100, 250, and 500), suggesting no interaction between sample size and situational effects. Similarly, the MI-PGM’s consistent accuracy was maintained irrespective of different sample sizes. On the other hand, we observed interactions between the analysis models and the magnitude of situational effects. Specifically, the impact of situational influences on reliability estimation was pronounced solely within the SI-PGM. These findings emphasize the advantage of using the MI-PGM when capturing reliability in the presence of at least a small degree of situational influences.

## Discussion

Our goal here was to integrate PGMs into the LST theory framework. This paper serves as an initial step towards capturing a more sophisticated measurement of trait changes and state variability in describing piecewise growth curves. SI-PGMs present a straightforward approach for examining changes that can be primarily attributed to trait effects. However, researchers might prefer MI-PGMs to incorporate both trait and situational effects and to improve the reliability of measurements.

The integration we provided holds several avenues for future research. For instance, the LST theory framework can be extended to more complex PGMs, including models with nonlinear (e.g., quadratic and exponential) growth patterns at each stage or multiple knots. For MI-PGMs in particular, the flexibility of incorporating both trait and state effects opens up further topics. One potential direction is measurement invariance testing, which can be used to assess whether the psychometric properties of measurements are consistent over time and hence capture pure state variability within segmented growth phases. Another topic is on modeling the methods effect to segregate indicator-specific effects from shared construct variance. Such an application may prove useful for identifying unique item contributions across separate growth phases.

## Supplemental Material

Supplemental Material - A Study of Latent State-Trait Theory Framework in Piecewise Growth Models Supplemental Material for A Study of Latent State-Trait Theory Framework in Piecewise Growth Models by Ihnwhi Heo, Ren Liu, Haiyan Liu, Sarah Depaoli, and Fan Jia in Applied Psychological Measurement

## Note

1 https://osf.io/dmtus/.
