# Supplementary material for: A Study of Latent State-Trait Theory Framework in Piecewise Growth Models
Source: Appl Psychol Meas. 2025 Jul 15;50(1-2):21–32. doi: 10.1177/01466216251360565 (PMC12264255; doi:10.1177/01466216251360565)
Supplement: Supplemental Material - A Study of Latent State-Trait Theory Framework in Piecewise Growth Models [file sj-pdf-1-apm-10.1177_01466216251360565.pdf]

Supplementary Materials

For the dissemination of the SI-PGM and MI-PGM and the replication of the simulation study, we have made the Mplus syntax and population values available to researchers as online supplementary materials through the Open Science Framework project (https://osf.io/dmtus/).
